# Transcriptional and bioinformatic analysis of GABA_A_ receptors expressed in oligodendrocyte progenitor cells from the human brain

**DOI:** 10.3389/fnmol.2023.1279232

**Published:** 2023-10-26

**Authors:** Berenice A. Gutierrez, José Manuel González-Coronel, Rogelio O. Arellano, Agenor Limon

**Affiliations:** ^1^Department of Neurology, Mitchell Center for Neurodegenerative Diseases, University of Texas Medical Branch, Galveston, TX, United States; ^2^Laboratorio de Neurofisiología Celular, Instituto de Neurobiología, Universidad Nacional Autónoma de México, Juriquilla, Mexico

**Keywords:** PDGFRA, fractional contribution, single cell, single nucleus, RNA-sequencing, oligodendrocyte, OPC, GABA receptor

## Abstract

**Introduction:**

Oligodendrocyte progenitor cells (OPCs) are vital for neuronal myelination and remyelination in the central nervous system. While the molecular mechanisms involved in OPCs’ differentiation and maturation are not completely understood, GABA is known to positively influence these processes through the activation of GABA_A_ receptors (GABA_A_Rs). The molecular identity of GABA_A_Rs expressed in human OPCs remains unknown, which restricts their specific pharmacological modulation to directly assess their role in oligodendrocytes’ maturation and remyelination.

**Methods:**

In this study, we conducted a transcriptomic analysis to investigate the molecular stoichiometry of GABA_A_Rs in OPCs from the human brain. Using eight available transcriptomic datasets from the human brain cortex of control individuals, we analyzed the mRNA expression of all 19 known GABA_A_Rs subunit genes in OPCs, with variations observed across different ages.

**Results:**

Our analysis indicated that the most expressed subunits in OPCs are α1–3, β1–3, γ1–3, and ε. Moreover, we determined that the combination of any α with β2 and γ2 is likely to form heteropentameric GABA_A_Rs in OPCs. Importantly, we also found a strong correlation between GABA_A_R subunits and transcripts for postsynaptic scaffold proteins, suggesting the potential postsynaptic clustering of GABA_A_Rs in OPCs.

**Discussion:**

This study presents the first transcriptional-level identification of GABA_A_R subunits expressed in human OPCs, providing potential receptor combinations. Understanding the molecular composition of GABA_A_Rs in OPCs not only enhances our knowledge of the underlying mechanisms in oligodendrocyte maturation but also opens avenues for targeted pharmacological interventions aimed at modulating these receptors to promote remyelination in neurological disorders.

## Introduction

Oligodendrocyte progenitor cells (OPCs) are among the most abundant glial cells distributed throughout the brain. These cells are known for maintaining the myelination of the central nervous system throughout an individual’s lifespan (Dawson et al., 2003; Trotter et al., 2010; Richardson et al., 2011; Gallo and Deneen, 2014; Fernandez-Castaneda and Gaultier, 2016). Under normal circumstances, acute myelin loss is compensated through a sequence of events involving the activation, proliferation, migration, and differentiation of OPCs into the damaged zone where myelin was compromised (van Tilborg et al., 2018; Baydyuk et al., 2020). It is suggested that this sequence begins with the involvement of GABA_A_ receptors (GABA_A_Rs) expressed in OPCs. When these receptors are activated by GABA—released at direct synaptic contacts with axons (Lin and Bergles, 2004; Jabs et al., 2005; Gallo et al., 2008)– it leads to membrane depolarization and an increase in cytosolic Ca^2+^ (Kirchhoff and Kettenmann, 1992; Arellano et al., 2016), thereby setting off the distinct stages of OPC maturation and remyelination (Cheli et al., 2016; Santiago González et al., 2017; Marisca et al., 2020). However, in some life-threatening conditions, including genetic disorders (leukodystrophies), traumatic brain injuries, and autoimmune diseases, there appears to be a decrease of response of OPCs to demyelination, which correlates with the clinical presentation of these disorders (Raabe et al., 2018; Butt et al., 2019; Gruchot et al., 2019; Huntemer-Silveira et al., 2020).

We propose that the control of GABA_A_Rs activity and its downstream signaling in OPCs may be critical for the development of drugs aimed at treating demyelinating diseases (Reyes-Haro et al., 2021). However, the stoichiometry of GABA_A_Rs in human OPCs remains unknown. GABA_A_R pentamers are commonly assembled from subunits of three different families, encoded by a pool of 19 genes (α1–6, β1–3, γ1–3, δ, ε, π, θ, ρ1–3), and they are usually comprised of two α, two β, and one γ or δ subunits (McKernan and Whiting, 1996; Petri et al., 2002). Through transcriptional and functional studies, we recently observed that most GABA_A_Rs in OPCs from neonate (P0-P14) rats and mice are most likely made up of α3β2γ1 subunits (Ordaz et al., 2021), and in one human dataset, we found that the most expressed subunits were α3, β1 and 3, γ1, γ2, and ε (Serrano-Regal et al., 2020), suggesting potential differences between humans and rodents. To better determine the potential diversity of GABA_A_Rs subunit expression in the human brain, we used multiple publicly available human RNA sequencing datasets, and we explored the likely stoichiometry of GABA_A_Rs in OPCs, their enriched molecular pathways and protein networks, their correlation with accessory proteins, and their potential differences in expression as a function of age.

## Results

### Gene expression yield differences of GABA_A_R subunits across datasets

We downloaded three single-cell (sc) and five single-nucleus (sn) RNA-sequencing datasets from the Gene Expression Omnibus (GEO) repository^1^ and the Allen Brain Map^2^ (Supplementary Table 1). We chose datasets derived from healthy cortical tissue (frontal and temporal cortex) from fetal (*n* = 8 subjects), pediatric (*n* = 9), adolescent (*n* = 1), and adult (*n* = 42) human populations. To identify OPCs among all other cell types, we selected cells expressing the platelet-derived growth factor α type receptor (*PDGFRA*), a marker enriched in OPCs (Pringle et al., 1992; Pringle and Richardson, 1993; Miller et al., 1999). In this selection, we found a total of 12,822 OPCs, with 11% (1,419 OPCs) expressing at least one of the 19 GABA_A_R subunits. More specifically, in adults, 20% (1,007/4,796) of *PDGFRA*+ cells expressed at least one GABA_A_R subunit, compared to 0.6% (20/3,086 OPCs) in adolescent, 6% (268/4,502 OPCs) in pediatric, and 28% (124/438 OPCs) in fetal populations (Supplementary Table 2).

Given that GABA_A_Rs are typically present in OPCs, the low percentage of GABA_A_R subunit expression was unexpected. We hypothesized that this might be related to different selection criteria among research groups during quality control. Consequently, we also performed an analysis in CellRanger using FASTQ files from four out of the eight datasets we obtained from the GEO repository and the Allen Brain Map (specifically PRJNA5776618, PRJNA673712, PRJNA589018, PRJNA674571). The FASTQ files for Jäkel et al. (2019) (GSE118257) and Hodge et al. (2019)^3^ were not publicly accessible. Although Darmanis et al. (2015) (PRJNA281204) and Lake et al. (2018) (PRJNA383372) had FASTQ files available, they were incompatible with CellRanger due to the unique UMI/Cell Barcode/Sequencing index used in their Drop-seq platform. From our analysis of the four compatible FASTQ datasets, we categorized OPCs as *PDGFRA*+ cells and examined their GABA_A_R subunit gene expression in the adult healthy control cortex. Interestingly, out of 635 OPCs (*PDGFRA*+ cells) we identified, 325 (51%) expressed at least one of the 19 GABA_A_R subunits (Supplementary Table 2). This contrasts with results from the same four datasets directly sourced from the GEO repository, where 4,002 OPCs were identified, but only 447 (11%) exhibited expression of any of the 19 GABA_A_R subunits (Supplementary Table 2). This data implies that selection criteria for quality control can significantly influence the percentage of recognized OPCs. However, it remains unclear whether this discrepancy affects the contribution of GABA_A_R subunit genes to OPCs.

**Figure 1:**
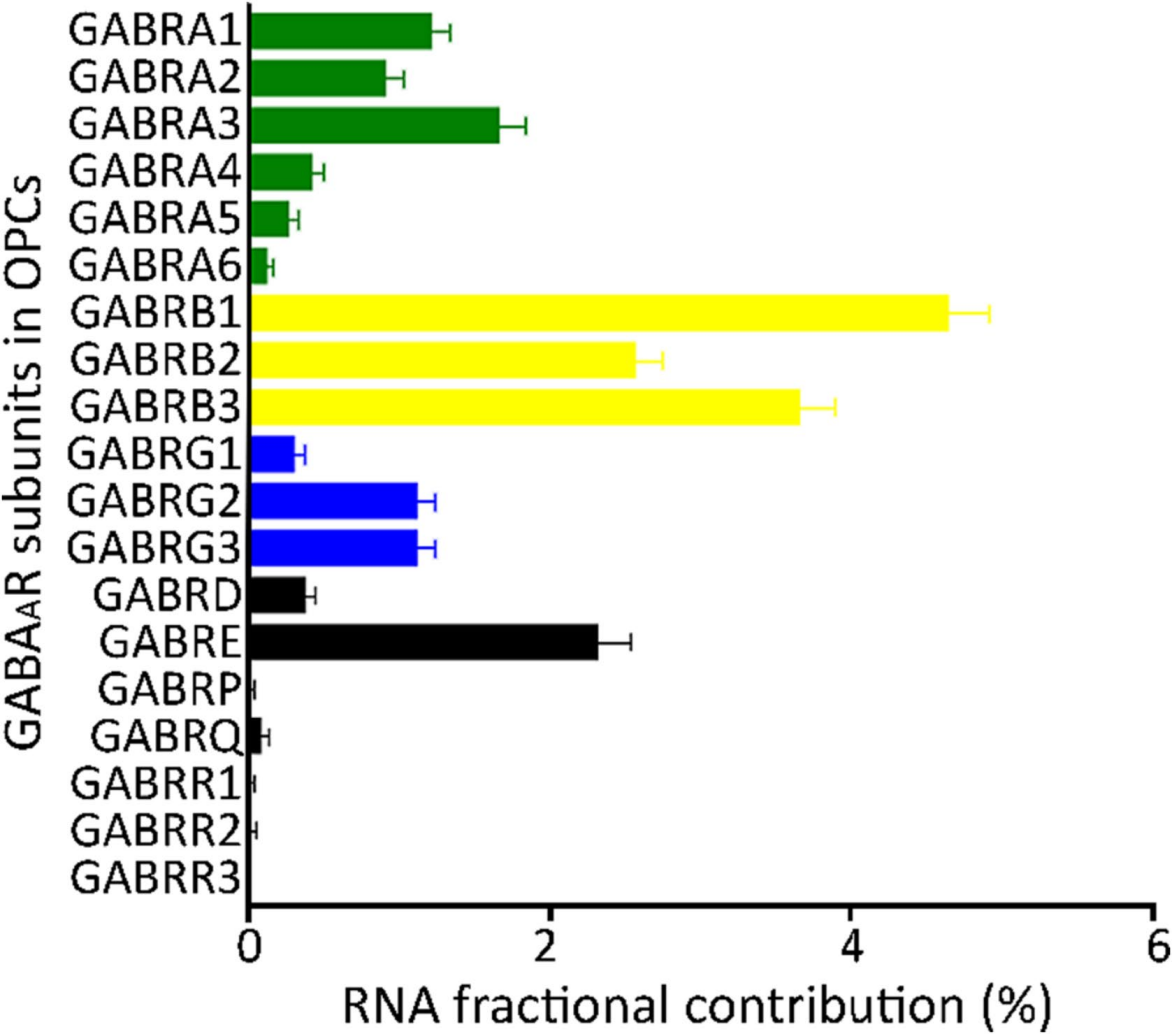
Fractional contribution of GABA_A_R subunit genes. Average fractional contribution (FC) of each GABA_A_R subunit gene, derived from 1,007 *PDGFRA*+ cells (OPCs) across 8 datasets from the human cortex (GSE138852, GSE155488, GSE67835, GSE118257, GSE140231, GSE160813, GSE97930, https://celltypes.brain-map.org/rnaseq). Bars depict the mean FC of GABA_A_R subunit gene across the eight datasets, and standard error (SE) is indicated for each bar.

To integrate the GABA_A_R subunits from all seven GEO datasets and one from the Allen Brain Map, we transformed the raw data expressed as unique molecular identifiers (UMIs) or FPKMs, into fractional contribution (FC), which is the percentage of the sum of the expression levels of each subunit gene in each cell over the sum of all 19 subunit genes. This metric reflects the contribution of each subunit to the total mRNA available to form GABA_A_Rs (Sequeira et al., 2019). The FC from each of the eight datasets indicated that the family of β subunits has the largest contribution, whereas ρ and π subunits have minimal contribution. The subunits α1/3 and γ2/3, showed more contribution compared to other subunits from their respective families (Figure 1A).

In our analysis of four individual GEO datasets, we observed variations in subunit contributions between them (Supplementary Figures 1A,C,E,G). This diverse contribution of different subunits across datasets suggests the existence of multiple GABA_A_R subunit stoichiometries in human OPCs. When using the FC analysis on these datasets with available FASTQ files, there were also noticeable differences in subunit contributions. However, in many instances, these contributions remained consistent (Supplementary Figures 1B,D,F,H). For the OPCs in these four FASTQ datasets, the most dominant GABA_A_R subunits were α1–3, β1–3, γ2, and δ (Supplementary Figure 2A). Similarly, when examining the same four datasets from the GEO repository, the primary contributors were α1–3, β1–3, γ2/3, and ε (Supplementary Figure 2B).

Importantly, single-cell data tends to be highly variable. The coverage level within a single cell is approximately 5%–40% of what is observed in bulk mRNA (Lun et al., 2016; Van den Berge et al., 2018; Choi et al., 2020; Ding et al., 2020). This disparity can largely be attributed to a significant number of cells that show little to no expression of certain subunits. Given these considerations, we incorporated two alternative methods to compute the FC (refer to “Materials and methods: Determination of FCs” for details): FC2 was derived from the expression level of each subunit gene relative to the sum of all 19 gene subunits per dataset, and FC3 was based on the percentage of cells expressing each subunit gene relative to the total *number of cells* (Supplementary Table 3). By employing these analytical approaches, we identified contributions of the majority of the 19 GABA_A_R subunit genes in human OPCs. Notably, subunits α1–3, β1–3, γ2/3, and ε showed the highest contributions (Figure 1; Supplementary Figures 3A,B). As a result, our validation indicates that for most subunits, any of the three FC methods can be reliably employed to normalize the expression levels across datasets.

**Figure 2:**
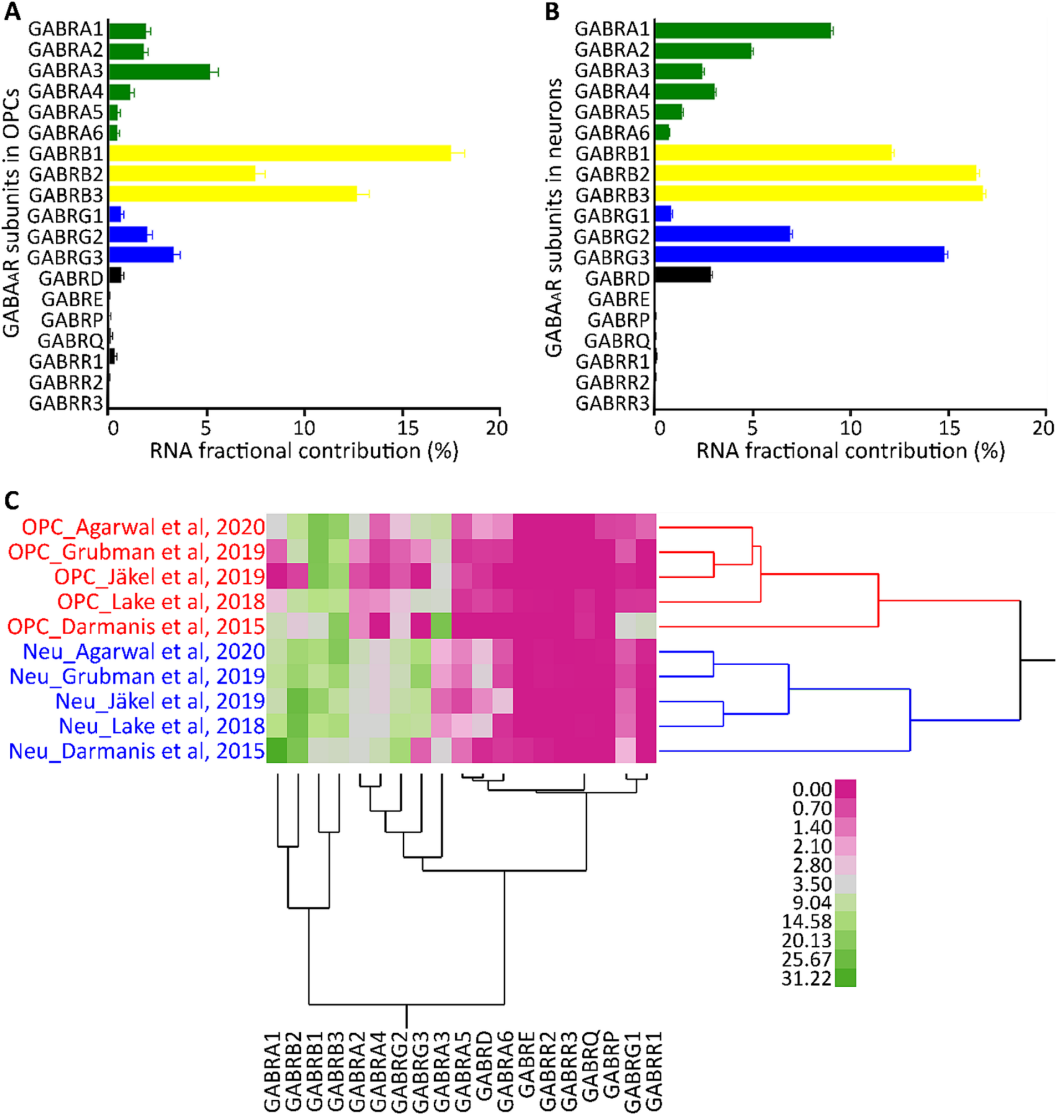
Segregation analysis of GABA_A_R subunit genes in OPCs and neurons. Average FC for each GABA_A_R subunit gene based on 2,431 barcoded OPCs **(A)** and 26,155 barcoded neurons **(B)** across five datasets from the human cortex (GSE14023, GSE67835, GSE138852, GSE118257, GSE97930). Bars indicate the mean FC of GABA_A_R subunit genes from the five datasets, and SE is shown for each bar. **(C)** Cell type segregation analysis, using the mean FC of each GABA_A_R subunit gene from the five datasets with barcoded OPCs and neurons. This is illustrated by a heatmap with a dendrogram using robust standardization, produced by JMP software (JMP, RRID:SCR_014242). GABA_A_R subunit gene names are found in the columns, while datasets (cell type_author) are listed in rows. Pink and green represent the abundance levels of GABA_A_R subunit genes.

The expression pattern of GABA_A_R subunits in cortical neurons has been well established (Sieghart and Sperk, 2002; Olsen and Sieghart, 2009). To understand if these patterns are consistent in different cell types, we compared OPCs and neurons (identified by their unique barcodes) from five GEO datasets (GSE14023, GSE67835, GSE138852, GSE118257, GSE97930). Our analysis revealed that both cell types have high contributions of subunits α1–4, β1–3, γ2/3, and δ. However, within the OPCs, α3, β1, and γ3 dominated their respective subunit families (Figure 2A). In contrast, neurons predominantly had α1, β2/3, and γ2/3 subunit contributions (Figure 2B), consistent with previous studies (Serrano-Regal et al., 2020; Ordaz et al., 2021). The contribution of other subunits was reduced in both cell types. To determine if each cell type possesses a distinct molecular signature of GABA_A_R subunits, we conducted a two-way hierarchical clustering analysis comparing OPCs and neurons. This was based on data from the five GEO datasets (GSE14023, GSE67835, GSE138852, GSE118257, GSE97930) which contains OPC and neurons, with cells identifiable by their unique barcodes. The analysis revealed two distinct clusters: one for OPCs and another for neurons across all datasets (Figure 2C). This underscores that each cell type has its unique molecular profile for GABA_A_Rs. We observed that the α, β, and γ subunits were particularly abundant, and these subunits consistently clustered together. While all subunits were present in OPCs, the α1–3, β1–3, and γ1–3 subunits contributed the most RNA and formed pronounced clusters. This suggests that the GABA_A_R stoichiometry in OPCs predominantly involves these subunit families.

### Putative stoichiometry of GABA_A_Rs in human OPCs

In neurons, it is well-known that GABA_A_Rs are heteropentameric complexes composed of two α, two β, and one γ subunit (McKernan and Whiting, 1996; Petri et al., 2002). Using this knowledge as a foundation, we examined the correlations formed by these subunits in the GABA_A_Rs of human OPCs.

**Figure 3:**
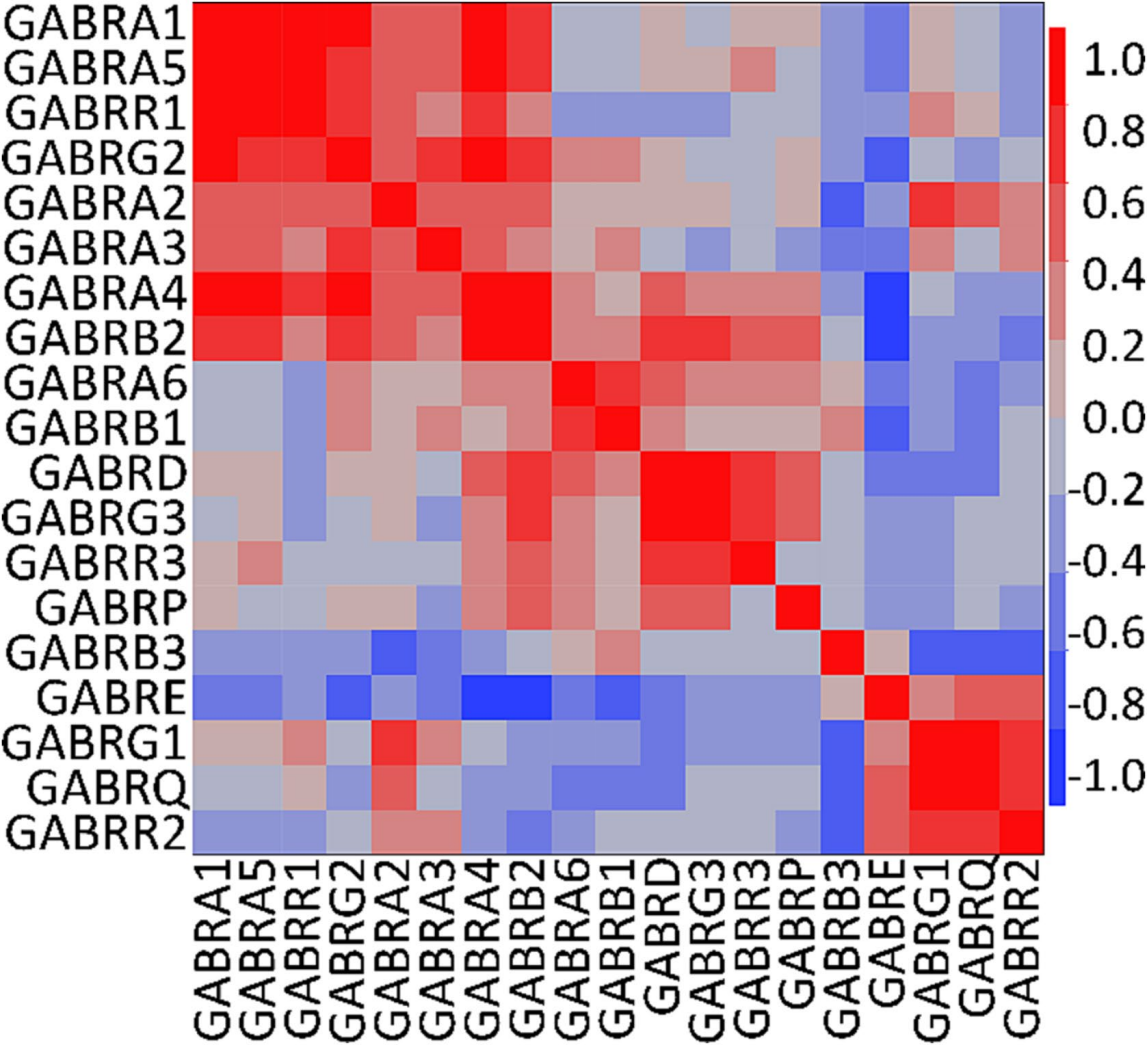
Correlation analysis of human GABA_A_R subunit genes in OPCs. Multivariate analysis using the mean of the fractional contribution of human GABA_A_R subunit genes from 1,007 PDGFRA+ cells (OPCs) across eight datasets of human cortex (GSE138852, GSE155488, GSE67835, GSE118257, GSE140231, GSE160813, GSE97930, https://celltypes.brain-map.org/rnaseq). The analysis was executed using JMP software (JMP, RRID:SCR_014242). Rows and columns are clustered based on correlation distance and average linkage. The correlation level of GABA_A_R subunit genes is represented by blue and red.

We conducted a multivariate analysis using the mean of the FC from each GEO and Allen Brain Map dataset for *PDGFRA*+ cells derived from healthy adult human cortical tissue. We found strong correlations among several subunits (Figure 3). As reported in previous literature, we observed correlations between α4 and α6 with the δ subunit which suggest the presence of receptors similar to the extrasynaptic receptors found in neurons (Mody, 2001). Further, we observed correlations between all α subunits with γ2, and between α4 and α6 with γ3. Complexes of α5 with γ2 have been observed in the hippocampal GABA_A_R in mice (Ghafari et al., 2017). However, correlations between α4 and α6 with γ subunits have not been previously described (Mody, 2001). Based on our observations and existing literature, potential GABA_A_R subunit combinations in OPCs may include α1β2γ1/2, α2β2γ1/2, α3β1γ1/2, and α5β2γ2.

### Biological processes of the GABA_A_R subunits

To further understand the cellular and biological processes that the expression of GABA_A_R subunits in OPCs might influence, we conducted a Gene Ontology (GO) analysis using all GEO and Allen Brain Map datasets. We identified genes with the strongest correlations to the GABA_A_R subunit genes and carried out the GO analysis on the first 1,000 genes that demonstrated a significant correlation (*p* < 4.2e-322; Supplementary Table 4).

**Figure 4:**
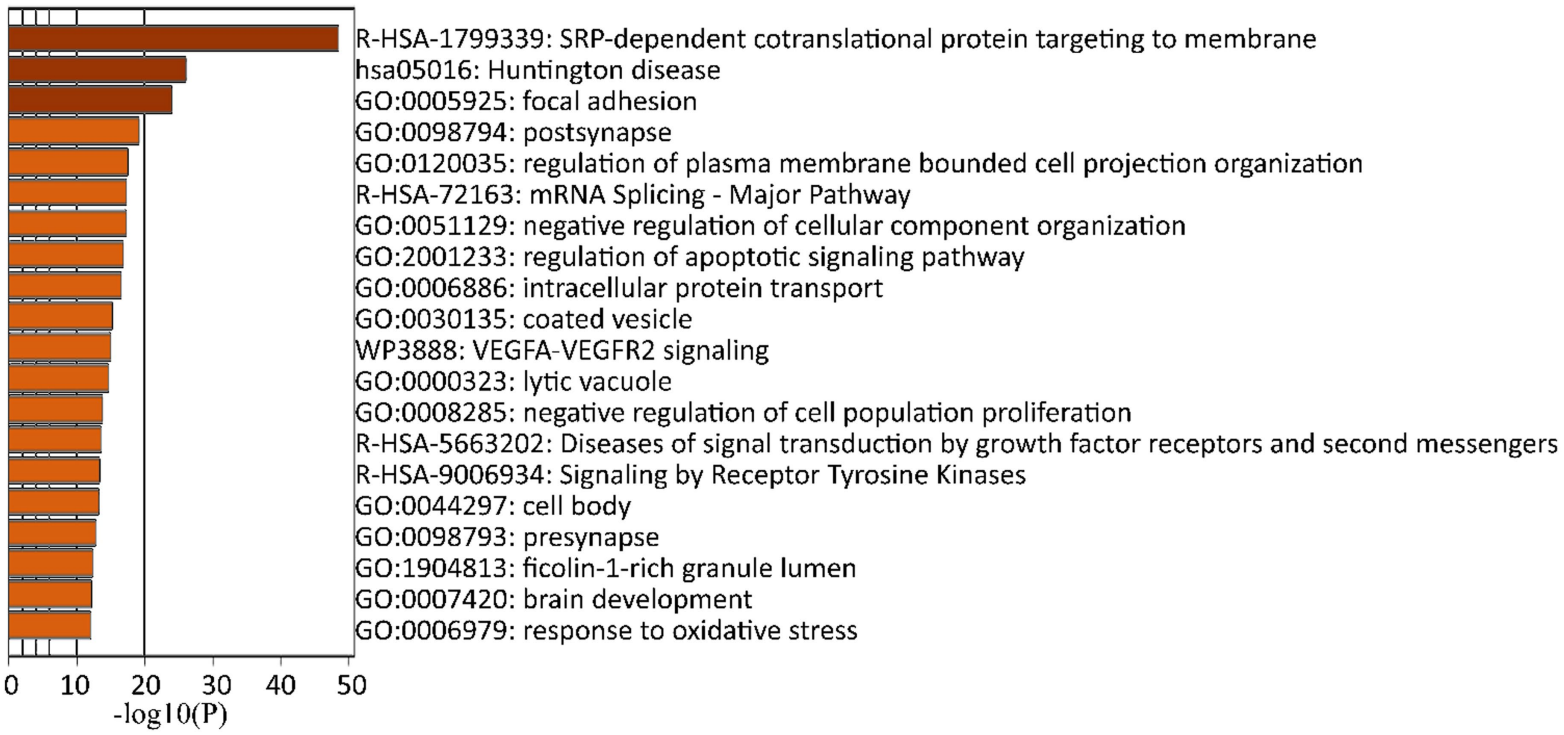
GO analysis of human GABA_A_R subunit genes in OPCs. A twenty most significantly (*p* ≤ 0.05) enriched GO terms in biological process, cellular components, and molecular functions impacted by GABA_A_R subunit genes using the Metascape GO enrichment platform. These terms were derived from the top 1,000 genes showing the strongest correlation with GABA_A_R subunit genes across eight datasets (GSE138852, GSE155488, GSE67835, GSE118257, GSE140231, GSE160813, GSE97930, https://celltypes.brain-map.org/rnaseq). All statistically significant values of the terms were transformed using a base 10 logarithm and are displayed as negative values. The terms are color-coded according to their respective *p*-values.

The analysis revealed that this set of genes are involved in neural pathways. Importantly, these genes contribute to the signal recognition particle (SRP)-dependent cotranslational protein targeting to the membrane, are implicated in Huntington’s disease, and are involved in focal adhesion and postsynapse pathways (Figure 4; Supplementary Tables 4, 5). Specifically, the gene encoding radixin (*RDX*), was found among the first thousand genes showing the strongest correlation with GABA_A_R subunit genes (*p* = 3.54293E-139; Supplementary Table 4). Additionally, robust correlations were found for other genes encoding scaffold proteins such as neuroligin 2 (*NLGN2*; *p* = 4.19956e-322), collybistin (*ARHGEF9*; *p* = 3.54293E-139), and GABA type A receptor associated protein like 1 (*GABARAPL1*; *p* = 3.69304E-139; highlighted in Supplementary Table 4). As these genes encode for scaffold proteins in postsynaptic membranes, their strong correlations with GABA_A_R subunit genes suggest “postsynaptic” clustering of GABA_A_Rs in OPCs. Surprisingly, despite its conventional association with GABAergic synapses, the correlation with gephyrin (*GPHN*) was not statistically significant (*p* = 0.5367362811).

### Differences of GABA_A_R subunits in OPCs during development

To assess age-related differences in the expression of GABA_A_R subunits, we used two GEO datasets (GSE155488 and GSE160813) spanning three age groups. We compared the expression levels of the GABA_A_R subunits in *PDGFRA*+ cells (OPCs) from fetal tissue derived from the telencephalon, pediatric tissue from the central area, and adult tissue from both the supratentorial region and the temporal cortex (Supplementary Table 1).

**Figure 5:**
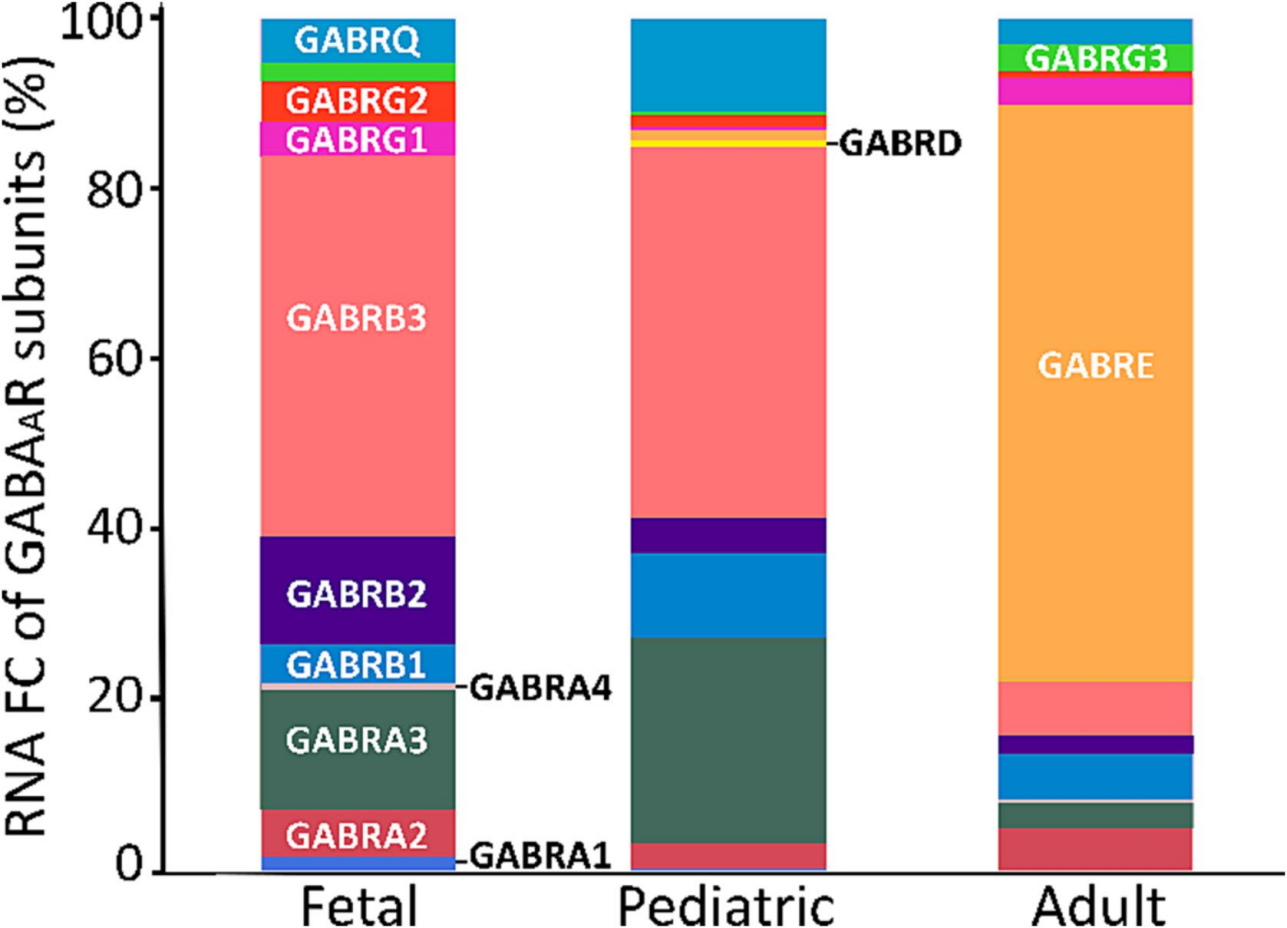
FC of GABA_A_R subunits across age span. A cross-sectional analysis of GABA_A_R subunits FC was conducted using 125 fetal, 268 pediatric, and 1,007 adult PDGFRA+ cells (OPCs) derived from two datasets of the human cortex (GSE155488 and GSE160813). The bars represent the mean percentage of RNA FC from both datasets for each GABA_A_R subunit.

From our analysis of the FC for each subunit, we observed that adults predominantly exhibited higher expression levels of the ε subunit. Furthermore, besides ε, the notable subunits in adults were α2, β3, and γ3. In contrast, the fetal and pediatric populations mainly expressed α3 and β3 (Figure 5).

## Discussion

To the best of our knowledge, this is the first report that aims to identify the most probable subunit combinations that constitute the GABA_A_R in human OPCs based on transcriptomic analysis. We found that while OPCs show varied expression of GABA_A_R subunits, the α, β, and γ subunits are predominantly expressed. However, among these expressed subunits, α1–3, β1–3, γ1–3, ε, and δ subunits demonstrate the highest FCs. Given the significant FC of various subunits within the same group (e.g., α1–3), it suggests the existence of multiple stoichiometries for the receptor. Notably, certain α, β, and γ subunits exhibited strong correlations in our computational analysis. Although these correlations do not directly indicate physiological interactions, they do highlight the need for further functional characterization. Building on prior experimental findings in rats, we expect that at least some of these receptor stoichiometries will be confirmed *in vivo*.

GABA_A_Rs in oligodendrocyte (OL) lineage cells respond similarly to the same pharmacological agents as neurons, indicating shared receptor subunits. However, as evidenced in rat OL electrophysiological studies (Arellano et al., 2016; Cisneros-Mejorado et al., 2020) they occasionally exhibit differential responses to specific drugs. For example, benzodiazepines such as diazepam potentiate GABA_A_Rs in both OL lineage cells and neurons (Möhler, 2002). Yet some β-carbolines like β-CCB, which inhibit the GABA-response of neurons, enhance GABA_A_Rs in OLs. Additionally, Zn^2+^ has no effect on neuronal GABA_A_Rs containing the γ2 subunit, but inhibits OL lineage cells, implicating the γ1 subunit (Ordaz et al., 2021). These differential responses confirm that each cell type has its unique GABA_A_R stoichiometry. Previous studies have proposed stoichiometries for GABA_A_Rs in rat OPCs based on their pharmacological profiles, highlighting the incorporation of α3, β2, and γ1 (Arellano et al., 2016; Ordaz et al., 2021). In contrast, our human transcriptomic analysis revealed strong correlations between all α subunits with both β2 and γ2, and a reduced correlation of α subunits with β1 and γ1. Two of our datasets showed high ε subunit expression levels (Allen Brain Map and GSE155488; Hodge et al., 2019; Perlman et al., 2020). The ε subunit can form a GABA_A_R pentamer subtype with two α and two β subunits in the brain and liver (Erlitzki et al., 2000). Intriguingly, this subtype is insensitive to the effects of intravenous anesthetic agents (Davies et al., 1997) and is significantly increased in the cerebellum of patients with schizophrenia, bipolar disorder, and major depression (Fatemi et al., 2013). However, our analysis did not find any correlations of ε with other subunits. Nevertheless, we found correlations of δ with α4/6 and β2, a combination consistent with the expression of extrasynaptic GABA_A_Rs. These extrasynaptic GABA_A_Rs (Jones et al., 1997; Peng et al., 2002), documented in electrophysiological studies on murine OLs cocultured with neurons, are of particular interest due to their strong sensitivity to neurosteroids (Belelli et al., 2002; Belelli and Lambert, 2005). These have been suggested to have a positive effect on myelination in various animal models (Ghoumari et al., 2003; Shaw et al., 2019).

As anticipated, the GO analysis showed GABA_A_R subunits’ involvement in neural pathways. Expression of GABA_A_Rs in OPCs has been well-documented (Bergles et al., 2010). Specifically, the GABA_A_Rs γ2 subunit has been suggested to be postsynaptic in OPCs from murines (Passlick et al., 2013; Balia et al., 2015; Orduz et al., 2015; McKenzie et al., 2018). We also found a strong correlation between GABA_A_Rs and ER translocation (R-HSA-1799339: SRP-dependent cotranslational protein targeting to membrane). Our findings confirm a previous transcriptomic analysis using induced pluripotent stem cell-derived O4+ OL lineage cells from an individual with the Parkinson’s Disease genetic variant SNCA, which demonstrated a correlation with SRP-dependent co-translational protein targeting to the membrane (Azevedo et al., 2022). This suggests that misfolding of GABA_A_R subunits could potentially disrupt the OPC maturation signaling cascade in various neurodegenerative diseases, including Parkinson’s Disease. This is also supported by evidence that alterations in human αSynuclein, the pathogenic hallmark of all synucleinopathies, impact OPC maturation (Ettle et al., 2014; Azevedo et al., 2022). Additionally, our GO analysis highlighted active participation in postsynaptic pathways, a finding we confirmed by detecting transcripts for postsynaptic proteins including radixin, neuroligin 2, collybistin, and GABA type A receptor-associated protein like 1. These findings align with a recent study by Patt et al. (2023). Importantly, our analysis revealed that gephyrin did not exhibit a significant correlation with GABA_A_R subunits in OPCs, setting it apart from the other identified postsynaptic proteins.

By analyzing the FASTQ file datasets using the CellRanger and Seurat pipelines, coupled with our quality control measures, we noted an increase in the number of *PDGFRA*+ cells compared to those in the GEO datasets. However, these differences did not impact the FC. The first three α, β, and γ subunits consistently showed the highest FCs. Hence, the quality control measures employed by the authors for the GEO datasets did not significantly affect the overall findings.

In our analysis of GABA_A_R subunits development, we relied on just two datasets (GSE155488 and GSE160813; Perlman et al., 2020; Fernandes et al., 2021) with RNA-seq data covering different ages. Yet, our findings showed the dynamic expression of GABA_A_R subunits in OPCs throughout various developmental stages. This suggests a persistently evolving role for these cells and likely functional significance during brain maturation. The ε subunit demonstrated a notably higher expression in adults compared to fetal and pediatric groups. This variation implies a potential shift in GABA_A_R function within OPCs as the brain matures. The increased representation of the ε subunit in adults might relate to distinct physiological roles or response dynamics unique to mature OPCs However, it is worth noting that of the original eight GEO adult datasets, only the one from Fernandes et al. (2021) (GSE160813) indicated substantial expression and contribution of the ε subunit. This warrants further analysis to determine the role, if any, of this subunit in GABA_A_Rs in adult human OPCs. Conversely, the dominant expression of α3 and β3 subunits in fetal and pediatric stages could emphasize their critical role during early brain development. In addition, we noted a distinctive pattern concerning the γ2 subunit, which was predominantly present in fetal populations, followed by pediatric populations, and was least prevalent in adults. This suggests a likelihood of decreasing γ2 subunits in adulthood. This finding aligns with a recognized characteristic of OPCs: as these cells age, they become less responsive to specific drugs like diazepam and zolpidem, indicating a reduction in the γ2 subunit of GABA_A_Rs (Vélez-Fort et al., 2010; Balia et al., 2015; Patt et al., 2023). Similarly, we observed a parallel trend with the δ subunit, where its presence decreases in adulthood. In contrast, the α3 subunit remained consistent across all age groups. Notably, previous research indicated that the α5 subunit becomes more prominent in the adult hippocampus, reflecting alterations in receptor subunits during development (Patt et al., 2023). However, in our datasets, α5 was not as prominent as α1–3 subunits. Despite this, our correlation analysis demonstrated that α5 is correlated with other subunits, suggesting the possibility of GABA_A_Rs containing α5 combined with other subunits in humans, with no discernible age-related changes. These observed shifts in receptor subunits indicate a change of GABA_A_Rs in OPCs across developmental stages.

Our study demonstrates that GABA_A_Rs in human OPCs likely have multiple stoichiometries due to the considerable variation in the expression of different subunits. The significant correlation we observed between subunits suggests the presence of several distinct receptor forms, each potentially contributing to the pharmacological profile of GABA_A_Rs in OPCs. These findings enrich our understanding of GABA_A_R diversity, emphasizing the need for further functional characterization of these receptors in OPCs from human. Understanding the specific compositions of GABA_A_Rs in OPCs could shed light on the physiological role of these cells in neurodevelopment and disease. For example, in the datasets we examined, the one provided by Jäkel et al. (2019) (GSE118257) contained RNA-sequencing data on patients with multiple sclerosis (MS), a severe demyelinating disorder. It would have been intriguing to investigate potential differences in GABA_A_Rs under such condition. However, this dataset only included 14 *PDGFRA*+ cells from active lesions of MS out of 295 total cells. This limited sample size lacked the statistical power necessary to discern alterations in GABA_A_R stoichiometry within OPCs during pathology. The scarcity of RNA-sequencing datasets from human tissues affected by demyelinating diseases currently restricts our ability to conduct a comprehensive analysis in this regard. Additionally, exploring a different scenario could be equally enlightening. For instance, studying musicians who have higher intracortical myelination (Kim and Knösche, 2016) might reveal if there are variations in GABA_A_R stoichiometry that contribute to enhanced myelination. This investigation could give us insights into the potential role of OPCs in neural plasticity and activation. Nevertheless, this avenue of research remains unexplored, leaving a promising area for future studies to unravel the complexities of the function of GABA_A_Rs in OPCs in diverse physiological and pathological contexts. Furthermore, it may lead the way for developing targeted therapies for demyelinating diseases, neurological disorders, and other conditions linked to oligodendrocyte dysfunction.

## Materials and methods

### Datasets

We retrieved three scRNA-sequencing and five snRNA-sequencing libraries from the Gene Expression Omnibus and Allen Brain Map repositories. The specimens used for sequencing were sourced either fresh from surgical resections or frozen from brain tissue banks, covering the frontal cortex, temporal cortex, visual cortex, and entorhinal cortex (refer to Supplementary Table 1). The sequencing data used in our study came from control fetal, pediatric, adolescent and adult subjects, with no known neurological or psychiatric illnesses. We extended the age range from fetuses to adults to explore the differences in subunit expression during development. For surgical specimens, we only included sequencing data derived from tissue devoid of pathological features. The datasets contained barcodes, genes, and matrix files, which we merged using R software. We sorted OPCs in each dataset by selecting *PDGFRA*+ cells, followed by selecting the GABA_A_R subunit genes and examining their expression levels. We used JMP software for these processes (JMP, RRID:SCR_014242).

### Determination of FCs

We normalized the different units (unique molecular identifier counts, fragments per kilobase million reads, and raw read values) found in the raw data by calculating the FC of individual subunits. The FC of each GABA_A_R subunit per cell type is the percentage of expression level of each subunit gene to the total pool of subunit genes within each cell type. For this, the sum of unique molecular identifier counts, fragments per kilobase million reads, and raw read values mapped reads in RNA-Seq data, of all 19 genes per cell type was 100% (Sequeira et al., 2019). Additionally, we explored the use of other two FC methods: For FC2, we obtained the percentage of expression level of each subunit gene over the sum of all 19 gene subunits per dataset. And for FC3, we obtained the percentage of number of cells expressing each subunit gene over the total number of cells. Thus, FC1 represents the mean ± SD of the FC for each dataset (Fc_d_), where Fc_d_ is the corresponding mean value of the FC in each cell (Fc_c_), FC2 represent the single subunit FC value for all the dataset and FC3 represents the probability of the subunit to be found in the dataset. The same methods were applied to neurons as a validating control group.

### Segregation analysis

We performed a two-way hierarchical clustering with the FCs of five datasets (GSE14023, GSE67835, GSE138852, GSE118257, GSE97930) featuring barcoded OPCs and neurons. JMP software facilitated this analysis, with data being robustly standardized.

### Correlation analysis

We conducted a multiple Pearson’s correlation analysis of the subunits using the mean of the FCs across all datasets. After generating co-clustering heatmaps for each combination of subunit gene expression values, we reorganized the subunits into representative clusters using JMP software.

### Gene ontology analysis

All datasets were included in this analysis. We defined OPCs as those cells positive for *PDGFRA*. We calculated the sum of all GABA_A_R subunits per cell across all the included datasets. Then, using JMP software, we performed a response screening test to compare the GABA_A_R subunit genes with the rest of the available genes, thereby predicting the best gene combinations. We selected the top 1,000 genes with the lowest *value of p* (cutoff *p* < 4.2e-322) from the resulting analysis and investigated them using the Metascape GO enrichment platform (Zhou et al., 2019). We designated *H. sapiens* for both species input and analysis. Enrichment analysis, encompassing pathways and processes, was undertaken using these ontology sources: GO Biological Processes, GO Cellular Components, KEGG Pathway, Reactome Gene Sets, Canonical Pathways, CORUM, WikiPathways, and PANTHER Pathway.

### FASTQ data analysis

We downloaded FASTQ data from four datasets available in this format (PRJNA5776618, PRJNA673712, PRJNA589018, PRJNA674571) using the SRAtoolkits library. The quality of the downloaded files was verified using FastQC software. The data were then uploaded and analyzed in CellRanger to align reads, generate feature-barcode matrices, and perform clustering and gene expression analysis. We employed the Seurat pipeline for quality control, using the following parameters: min.cells = 3, min.features = 200, subset = nFeature_RNA < 3,000 and percent.mt < 5. These excluded cells expressing more than 5,000 genes and those with at least 500. It also filtered out cells where mitochondrial transcripts accounted for more than 5% of the total transcripts. Subsequently, we normalized the data using the command NormalizeData (seurat, normalization.method = “LogNormalize”) in Seurat. Following this quality control process, we identified OPCs as *PDGFRA*+ cells and examined the expression of their GABA_A_R subunit genes.

### Statistical analysis

We compared the means of all groups for continuous variables. For nonparametric comparisons, we applied the Wilcoxon test for multiple pairwise comparisons and the Wilcoxon/Kruskal-Wallis test for simple comparisons. We set statistical significance at *p* < 0.05.

## Data availability statement

The datasets presented in this study can be found in online repositories. The names of the repository/repositories and accession number(s) can be found in the article/Supplementary material.

## Author contributions

BAG: Data curation, Formal analysis, Investigation, Methodology, Software, Visualization, Writing – original draft, Writing – review & editing. JG-C: Investigation, Software, Writing – review & editing, Methodology. ROA: Conceptualization, Data curation, Formal analysis, Funding acquisition, Investigation, Methodology, Project administration, Resources, Software, Supervision, Validation, Visualization, Writing – original draft, Writing – review & editing. AL: Conceptualization, Data curation, Formal analysis, Funding acquisition, Investigation, Methodology, Project administration, Resources, Software, Supervision, Validation, Visualization, Writing – original draft, Writing – review & editing.

## Footnotes

^1^ https://www.ncbi.nlm.nih.gov/gds

^2^ https://portal.brain-map.org/atlases-and-data/rnaseq

^3^ https://celltypes.brain-map.org/rnaseq
