# Supplementary figures and images for: Transcriptional and bioinformatic analysis of GABAA receptors expressed in oligodendrocyte progenitor cells from the human brain
Source: Front Mol Neurosci. 2023 Oct 26;16:1279232. doi: 10.3389/fnmol.2023.1279232 (PMC10637375; doi:10.3389/fnmol.2023.1279232)

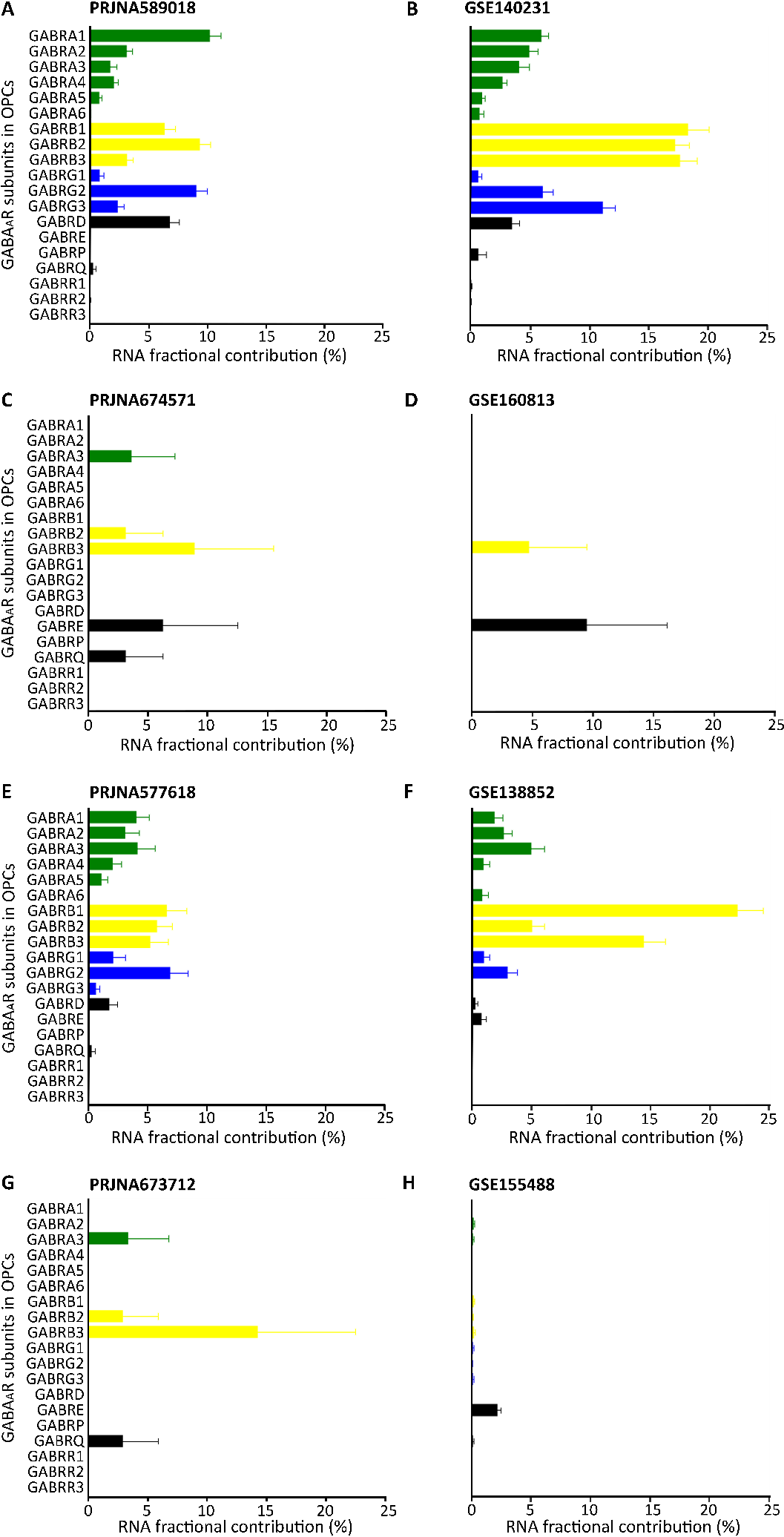

Supplement: Supplementary file 2 [file Image_1.tif]

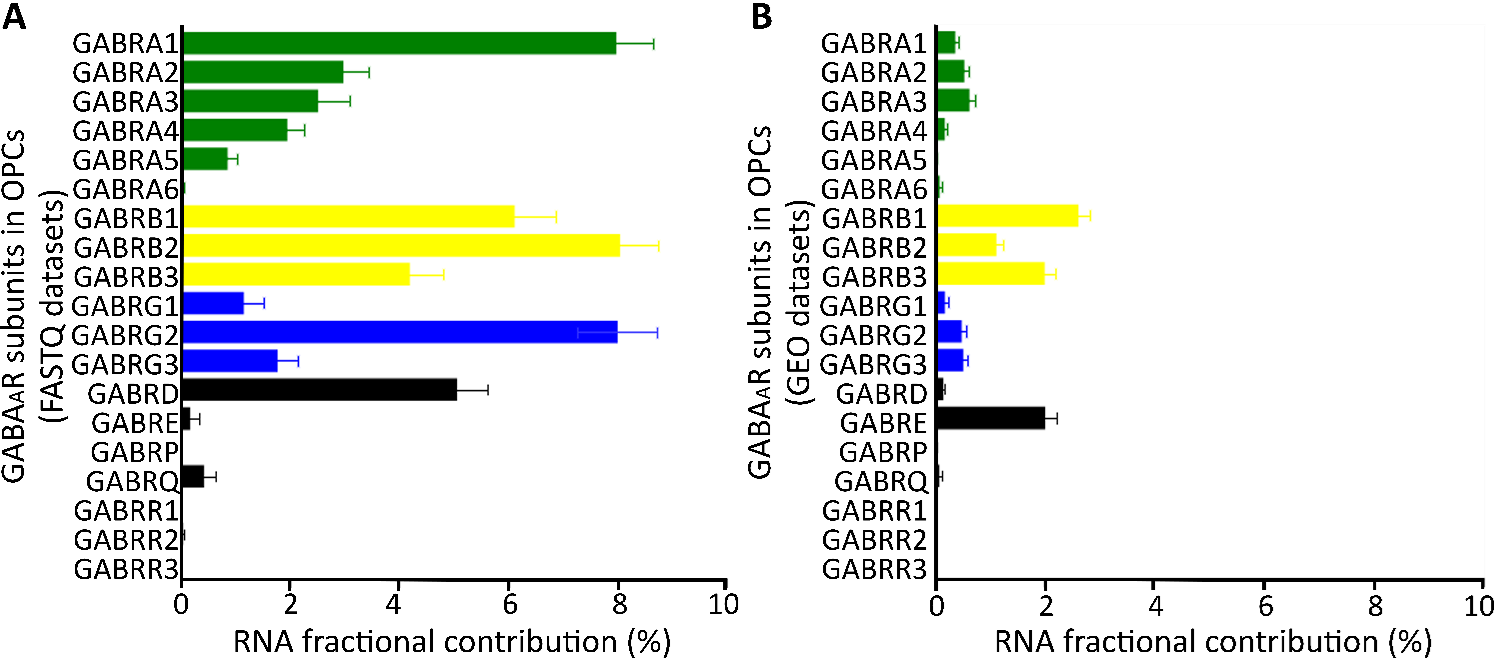

Supplement: Supplementary file 3 [file Image_2.tif]

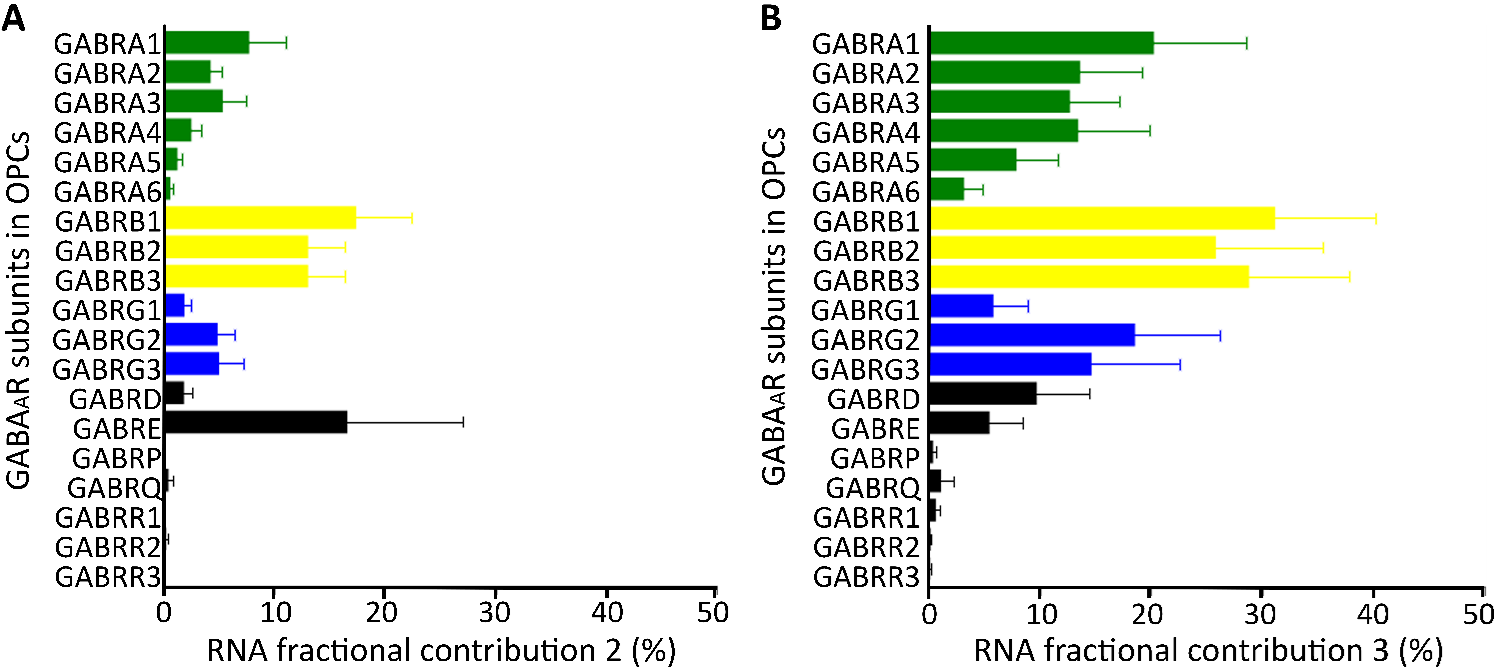

Supplement: Supplementary file 4 [file Image_3.tif]
